# Diagnostic accuracy of LiquidArray MTB-XDR VER1.0 for the detection of *Mycobacterium tuberculosis* complex, fluoroquinolone, amikacin, ethambutol, and linezolid susceptibility

**DOI:** 10.21203/rs.3.rs-4841978/v1

**Published:** 2024-08-05

**Authors:** Theron Grant

**Affiliations:** Stellenbosch University

**Keywords:** LiquidArray MTB-XDR, drug susceptibility testing, molecular diagnostics, tuberculosis, amikacin, ethambutol, linezolid, fluoroquinolones

## Abstract

Drug susceptibility testing (DST) is essential for effectively starting people on effective tuberculosis (TB) regimens. No accuracy data exists for the new high-throughput LiquidArray MTB-XDR (LA-XDR) test, which detects *Mycobacterium tuberculosis*complex (MTBC) and susceptibility to the fluoroquinolones, amikacin, ethambutol, and linezolid (the latter two drugs have no rapid molecular DSTs available). We enrolled (n=720) people with presumptive TB who provided two sputa for Xpert MTB/RIF Ultra and culture (MTBC reference standard). Phenotypic DST and Sanger sequencing served as a composite reference standard. Manual FluoroLyse and automated GenoXtract-fleXT (fleXT) DNA extraction methods were compared. For MTBC, LA-XDR using fleXT-extracted or FluoroLyse-extracted DNA had similar sensitivities (85–87%; which improved upon eluate retesting) and specificities (99%). Drug susceptibility sensitivities varied: 94% (86, 98) for fluoroquinolones, 64% (45, 80) for amikacin, and 88% (79, 93) for ethambutol (specificities 97–100%). LA-XDR detected 86% (6/7) phenotypically resistant linezolid isolates. LA-XDR with fleXT had indeterminate proportions of 8% (21/251) for fluoroquinolones, 1% (2/251) for ethambutol, 25% (63/251) for amikacin, and 37% (93/251) for linezolid. In a hypothetical population of 100 smear-negative fluoroquinolones-resistant cases, 24% (24/100) could be missed due to an unsuccessful result (1 fleXT error and, for LA-XDR, 2 invalid results, 15 MTBC-negative, 6 fluoroquinolone-indeterminate, 1 false-susceptible). LA-XDR met the minimum WHO target product profile for a next-generation sputum-based moderate complexity DST with high sensitivity for fluoroquinolones and ethambutol resistance, moderate sensitivity for amikacin resistance, and promise for linezolid resistance, for which more data are needed. Improved MTBC detection would reduce missed resistance.

## Introduction

Tuberculosis (TB) remains a devastating global health emergency, with an estimated 7.5 million new cases and 1.3 million deaths in 2022, and an estimated 410,000 multidrug-resistant (MDR) TB cases were reported^1^. The COVID-19 pandemic exacerbated TB management leading to underdiagnosis^1^. Between 2019 and 2022, there was a 15% decrease in drug-resistant (DR)-TB cases potentially obscured by COVID-19^2,3^. In 2022, the World Health Organization (WHO) endorsed a 6-month treatment regimen that incorporates fluoroquinolone (FQ) and linezolid (LZD) for MDR/RR-TB, replacing the longer 9-month or 18-month options^4^.

Current rapid molecular WHO-approved assays face limitations in detecting ethambutol (EMB) resistance, newer and repurposed drugs such as LZD resistance as the two can only be targeted by sequencing or phenotypic drug susceptibility testing (pDST)^5,6^. LiquidArray MTB-XDR VER1.0 (LA-XDR) assay (Bruker/Hain Lifescience GmbH, Nehren, Germany) is a high-throughput (24–94 tests within 3–5 hours) centralised DST. It is a successor to the MTBDR*sl* line probe assay^7^, integrates within laboratory information systems facilitating rapid results dissemination and, by minimising hands-on manipulation^8^ (only required during initial instrument setup and PCR plate transfer), reduces contamination risk^9^.

LA-XDR utilises the same DNA extraction platform as the WHO-recommended moderate complexity FluoroType MTBDR Ver2.0^10,11^, detects *Mycobacterium tuberculosis* complex (MTBC), and mutations conferring resistance to FQ in *gyrA* and *gyrB*, amikacin (AMK) in *rrs*, EMB in *embB*, and LZD in *rplC*, and *rrl* genes in both sputum and isolates^5,12–14^. It’s rapid DST, including for drugs for which there are no rapid tests like LZD and EMB offering a crucial component for effective TB management.

Undiagnosed resistance enhances the risk of selection of untreatable strains^13^, in other words, especially as rapid tests for new drugs do not yet exist, one of the best ways to protect them is to maximise the number of effective drugs (for example, fluoroquinolone resistance often precedes bedaquiline resistance^15^). This is the first evaluation of LA-XDR diagnostic performance in MTBC and resistance detection using two distinct DNA extraction methods, the manual FluoroLyse and automated GenoXtract fleXT (fleXT).

## Materials and Methods

### Study population.

Between 15 January 2020 and 23 May 2022, 720 de-identified sputa from ≥ 18 people with presumptive TB were collected from the National Health Laboratory Services (NHLS), a programmatic government laboratory in Cape Town, South Africa [92% (659/720)] and the FIND Biobank^16^ [8% (61/720)].

### Study Samples group.

Group A samples consisted of 87% (572/659) of NHLS samples from individuals with presumptive TB following Xpert MTB/RIF Ultra (Ultra) testing irrespective of smear status (**Supplementary Fig. 1**). 13% (87/659) of samples from the NHLS were delegated to Group B were Ultra rifampicin-resistant with either FQ-resistance or AMK-resistance confirmed by either Genotype MTBDR*sl* (MTBDR*sl*) or pDST at Stellenbosch University (**Supplementary Methods**). Group C samples (**Supplementary Fig. 2**) all from FIND consisted of 8% (61/720) of sputa and corresponding isolates with specific resistance confirmed by pDST or whole genome sequencing. Seven LZD phenotypic-resistant isolates from the Stellenbosch University biobank were included.

### FluoroLyse and GenoXtract fleXT DNA extraction.

DNA was extracted from sputa sediments and isolates using both the manual FluoroLyse and automated fleXT platform with GenoXtract X2 Extraction VER1.0 (Bruker/Hain Lifescience GmbH, Nehren, Germany) according to the manufacturer’s instructions^17^. A sample consisting of saline was included as a negative control, as well as the provided positive control. At the end of extraction, fleXT reports if the processing was successful or not (reported as an error with code).

### LiquidArray MTB-XDR testing.

MTBC and drug susceptibility detection was done using the FluoroCycler XT-96 (Bruker/Hain Lifescience GmbH, Nehren, Germany) instrument. PCRs were set up by the fleXT instrument using DNA extracted using fleXT. A manual PCR reaction was set up with 20 μl of FluoroLyse-extracted DNA added to a pre- mix of 14 μl AM-B and 6 μl AM-A solution per sample. Automated results interpretation was done by the FluoroSoftware XT-IVD (**Supplementary Fig. 3**). The LA-XDR test results can be categorised into successful (MTBC detected with DST-determinate; MTBC not detected), MTBC detected with DST-indeterminate, invalid and error (applicable to samples extracted with fleXT only).

### MGIT960 and phenotypic DST

pDST was done on Group A isolates if the index assay identified resistance. Group B underwent pDST for all drugs for LA-XDR detects resistance, while (**Supplementary Methods**) Group C only had LZD pDST done (samples were already characterised for FQ, AMK and EMB resistance by either WGS or pDST). pDST used the following WHO critical concentrations of kanamycin 5μg/ml, EMB 5μg/ml, ofloxacin 2μg/ml, moxifloxacin 2μg/ml, AMK 5μg/ml, and LZD 1μg/ml^18^. Negative control was done using the H37Rv (NC_000962) pan-susceptible MTBC strain.

#### Sanger sequencing.

Sanger sequencing was performed for all LA-XDR DST targets for all isolates from Group B MTBC-positive by LA-XDR. For Group C, sequencing was done on isolates for targets associated with LZD-resistance. Primers (Supplementary Table 1) for all key targets included FQ (*gyrA*, *gyrB*), AMK (*rrs*)^19^, EMB (*embB*)^20^, and LZD (*rrl*, *rplC*)^21^. FASTA files were aligned to the reference sequence of H37Rv (NC_000962) using BioEdit version 7.2.5 (https://bioedit.software.informer.com/7.2/).

#### Discrepant result analysis.

Repeat testing was done with remnant DNA to resolve result discrepancies, including false-negatives and false-positives detected by LA-XDR using FluoroLyse- or fleXT-extracted DNA. The repeat results were not included in the main analyses but are shown in **Supplementary Tables 2–3**.

### Data Analysis.

Statistical analysis, conducted with Stata 18.0 (StataCorp LP, Texas, USA), involved a two-sample test of proportions. Primary analyses assessed sensitivity and specificity for MTBC using a culture reference standard in Group A and B samples. To assess differences in sensitivity and specificity for the two DNA extraction methods, a McNemar test for paired data was done. P-values ≤ 0.05 were considered significant.

#### Ethics statement.

This study was done according to relevant guidelines and regulations, approved by the Stellenbosch University Health Research Ethics Committee (N16/04/045 and N09/11/296) and Western Cape Health Research Committee (2016RP18637). Permission was granted to access anonymised to-be-discarded residual samples collected in routine diagnostic practice with waived informed consent.

## Results

### Study population characteristics.

The median age was 39 years (18, 82) with women constituting 39% (278/713) of participants. 16% (113/713) were people living with HIV, while 41% (295/713) had an unknown HIV status (Table 1). Individuals with a history of previous TB constituted 14% (97/713). Of the samples, 44% (313/713) were culture-positive, with 24% (160/713) smear-positive.

### Study samples.

**Figure 1:**
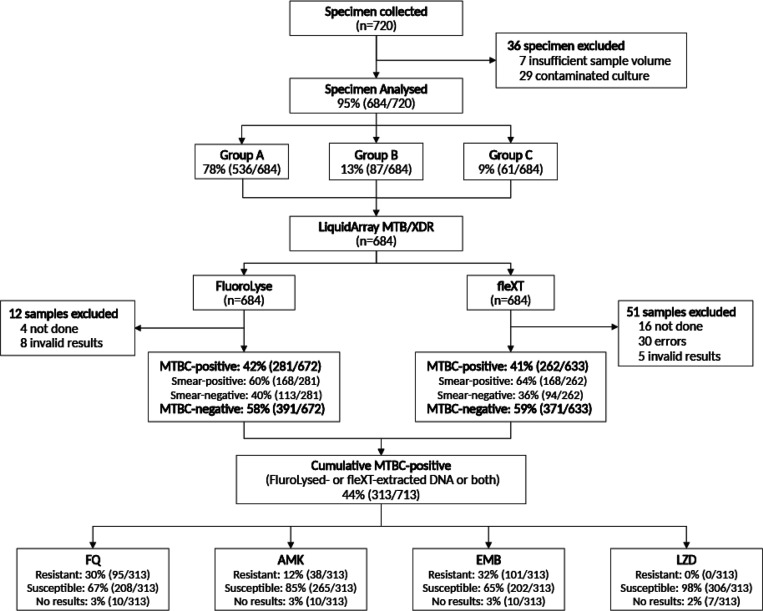
LiquidArray MTB/XDR performance proportion including participant enrolment and exclusions, drug susceptibility results from MTBC-positive samples. Out of 720 participants, 36 sputum samples were excluded due to insufficient volume or contamination. Group A (Ultra MTBC-negative and MTBC-positive (rifampicin-susceptible)) constituted 78%, while Group B made up 13% (rifampicin-resistant with resistance to FQ and/or AMK). Among the 684 analysed, 42% were MTBC-positive, with 60% of these being smear-positive. Cumulatively 313/684 MTBC-positive sputa by LA-XDR (FluroLysed- or fleXT-extracted DNA or both) were evaluated for drug susceptibility. Abbreviations: AMK, amikacin, EMB, ethambutol, fleXT, GenoXtract fleXT, FQ, fluoroquinolones, LZD, linezolid, MTBC, *Mycobacterium tuberculosis* complex.

Between 15 January 2020 and 23 May 2022, 720 sputa samples were collected (Fig. 1). 36 sputa were initially excluded because of insufficient volume (n = 7) or contaminated cultures (n = 29).

### Error, invalid, and indeterminate results from fleXT and LA-XDR

DNA extraction: Four people did not have sputum extracted using FluoroLyse and 16 people did not have sputa extracted using fleXT. fleXT had an error proportion of 4.5% (30/668) for sputum compared to 2.2% (7/322; p = 0.045) for isolates (**Supplementary Tables 4**).

Invalid MTBC detection: LA-XDR had a low invalid proportion in sputum testing of 1.3% (8/619) for FluoroLyse and 0.8% (5/607; p = 0.391) for fleXT. On repeat testing using left-over DNA, 100% (8/8) of invalid sputum FluoroLyse extracts became valid while 80% (4/5) by fleXT remained invalid. No invalid results occurred for testing on isolates.

DST Indeterminate: LA-XDR with fleXT DST using sputum, an indeterminate proportion of 38% (25/251) for AMK, 8% (21/251) for FQ and 1% (2/251) for EMB were observed. LZD DST on sputum had a indeterminate proportion of 51% (141/227) for FluoroLyse and 37% (93/251; p = 0.001) for fleXT.

#### LA-XDR performance compared to the composite reference standard.

**Figure 2:**
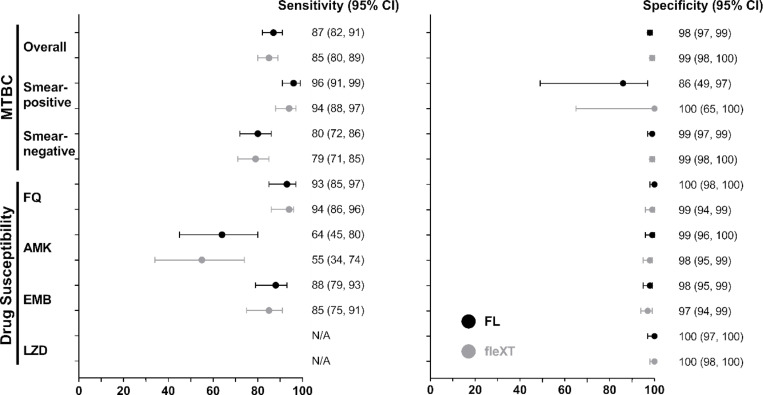
Forest plots showing diagnostic accuracy estimates variations (with 95% confidence intervals) for LiquidArray MTB/XDR on sputum samples. The data is stratified by DNA extraction method (FluoroLyse vs GenoXtract fleXT platform). MTBC detection estimates of smear-positives demonstrated slightly higher accuracy than for smear-negatives. Drug susceptibility performance estimates for AMK were lower than for FQ, EMB and LZD. However, the LZD sensitivity could not be determined due to no LZD resistance samples in the cohort. Abbreviations: AMK, amikacin; CI, Confidence Interval; EMB, ethambutol; FL, FluoroLyse; fleXT, GenoXtract fleXT; FQ, fluoroquinolones; LZD, linezolid; MTBC, *Mycobacterium tuberculosis*complex; N/A, not applicable;

MTBC detection: Overall sensitivity was 87% (95% CI: 82, 91) and 85% (80, 89; p = 0.528) by FluoroLyse and fleXT, respectively (Table 2). In smear-positive sputa extracted using FluoroLyse, sensitivity was 96% (91, 99) and specificity 86% (49, 97), compared to fleXT with a sensitivity of 94% (88, 97; p = 0.494) and a specificity of 100% (65, 100; p = 0.305). In smear-negative sputa, sensitivity was 80% (72, 86), with 99% (97, 99) specificity for FluoroLyse, while for the fleXT, sensitivity was 79% (71, 85; p = 0.839) and specificity 99% (97, 100; p > 0.999) (Fig. 2). Retesting false-negative FluoroLyse-extracted DNA eluates detected MTBC in 50% of smear-positive (2/4) and smear-negative (18/36) samples whereas retesting using fleXT-extracted eluates resulted in no MTBC detection in smear-positive (0/6) but 20% of smear-negatives (8/41). Using isolates, sensitivity was 98% (96, 99) by FluoroLyse compared to 99% (97, 100) for fleXT with specificity estimates of 93–100% for both methods. Three out of five isolates missed by LA-XDR were non-tuberculous mycobacteria by sequencing of *rrs* (*Mycobacterium intracellulare*, *Mycobacterium avium*, *Mycobacterium elephantis*), perhaps indicating mixed infection.

Fluoroquinolones: LA-XDR showed a sensitivity of 93% (95% CI: 85, 97) and 94% (86, 98) using FluoroLyse and the fleXT, respectively, with a specificity of 100% (98, 100) for FluoroLyse and 99% (96, 100) for fleXT (Table 3) on sputa. Using isolates, sensitivity was 92% (85, 94) and 92% (85, 96) for FluoroLyse and fleXT, respectively with a specificity of 100% (97, 99) for both. LA-XDR on FluoroLyse-extracted DNA showed comparable performance to MTBDR*sl* with a sensitivity of 92% (80, 97) vs 90% (76, 96; p = 0.758) on sputum and 93% (84, 97) vs 88% (77, 94; p = 0.359) on isolates (**Supplementary Table 5**). Based on successful results, in a hypothetical population of 100 smear-negative FQ resistance cases using fleXT (**Supplementary Table 6**), 24% (24/100) could be missed due to 1 fleXT error and, for LA-XDR, 2 invalid results, 15 MTBC-negative, 6 FQ-indeterminate, and 1 false-susceptible due to the sensitivity decrement.

Amikacin: LA-XDR showed a sensitivity of 64% (95% CI: 45, 80) and 55% (34, 74) using DNA extracted by the FluoroLyse and the fleXT platform respectively with a specificity of 99% (96, 100) for FluoroLyse versus 98% (95, 99) for fleXT in sputum samples. In isolates, sensitivity improved to 69% (95% CI 54, 81) for FluoroLyse compared to 72% (56, 84; p = 0.771) for fleXT with a specificity of 100% (98, 100) for both platforms. The assay demonstrated comparable performance to MTBDR*sl* with a sensitivity of 71% (45, 88) on sputum and 74% (54, 88) on isolates. In a hypothetical population of 100 smear-negative AMK resistance cases using fleXT, 53% (53/100) could be missed due to 1 fleXT error and, for LA-XDR, 2 invalid results, 15 MTBC-negative, 31 AMK-indeterminate, and 4 false-susceptible.

Ethambutol: LA-XDR had a sensitivity of 88% (95% CI: 79, 93) with FluoroLyse and 85% (75, 91) for fleXT with a specificity of 98% (95, 99) for FluoroLyse and 98% (95, 99) for fleXT. In isolates, sensitivity was 90% (83, 95) and 91% (76, 92) for the FluoroLyse and fleXT respectively with a specificity of 100% (97, 100) for both DNA extraction methods. In a hypothetical population of 100 smear-negative EMB resistance cases using fleXT, 23% (23/100) could be missed due to 1 fleXT error and, for LA-XDR, 2 invalid results, 15 MTBC-negative, 1 EMB-indeterminate, and 4 false-susceptible.

Linezolid: The assay exhibited a specificity of 100% (95% CI: 97, 100) for both methods. However, sensitivity could not be precisely assessed due to few resistant cases. LA-XDR detected 86% (6/7) of phenotypic-resistant isolates, with one being susceptible. All isolates with resistance detected by LA-XDR had the C-154-R *rplC* resistance-associated variant^22^.

## Discussion

Our data shows that using sputum, LA-XDR has: 1) high sensitivity for MTBC detection using either DNA method, 2) comparable FQ and AMK sensitivity and specificity to MTBDR*sl*, 3) high sensitivity and specificity for EMB resistance and 4) potential as a rule-in test for LZD resistance detection, particularly in where the *rplC* C-154-R variant is most frequent. Additionally, LA-XDR has relatively low indeterminate rates, however, due to suboptimal sensitivity for MTBC (~ 80% in smear-negatives), at least 20% of people will not have a susceptibility result generated.

LA-XDR sensitivity for MTBC (87%) is comparable to that of other WHO-approved high throughput, moderate complexity NAAT platforms (Abbott RealTime MTB-RIF/INH, Roche COBAS MTB-RIF/INH, BD MAX MDR-TB and FluoroType MTBDR)^9,23^. As expected, sensitivity in smear-negative specimens was diminished (80% vs. 96% for smear-positives), meaning that 20% (28/137) of smear-negative TB cases will be missed (and therefore, due to non-detection of MTBC DNA by LA-XDR, not have resistance or susceptible results generated, potentially resulting in missed resistance). Our results indicate that retesting smear-negative samples almost half the time leads to successful MTBC detection, which is valuable information for refining diagnostic algorithms. Additionally, if LA-XDR is employed as a reflex test following an initial positive TB screening, we recommend repeating the test using the remaining DNA extract.

Sputum FQ and AMK susceptibility sensitivity and specificity were comparable to those for MTBDR*sl* and like those for Xpert MTB/XDR^6,24,25^. FQ sensitivity (94%, independent of DNA extraction method) met the WHO target product profile (TPP) of > 90% sensitivity^26^. LA-XDR is promising in detecting EMB resistance, with sensitivity and specificity estimates of 88% and 98%, respectively. This is important because EMB is included in the management of MDR-TB in most clinical settings. The LA-XDR is the only option for programmes to do EMB DST and will fill the existing diagnostic gap as there is a high EMB resistance prevalence among MDR-TB^27^.

LZD is a key WHO group A drug regimen, LA-XDR has 100% specificity which makes it a valuable tool for ruling out LZD resistance. The test accurately detected the *rplC* mutation^5,28^ in phenotypic-resistant isolates and detected the most resistant specimens, demonstrating its potential for LZD susceptibility testing, especially in the context of no other commercially available molecular tools for susceptibility testing for this drug. On sputa samples, more than half (141/227) had indeterminate LZD results, possibly due to a high detection call threshold or unclear variant significance.

We demonstrated low AMK sensitivity estimates (64%), likely due to selection bias in FIND Biobank samples from Eastern Europe with the prevalent C-14-T *eis* promoter variant^22^ undetected by LA-XDR. WHO classifies the C-14-T *eis* promoter mutation as provisionally associated with resistance, it can only confer resistance if linked to a functional *eis*^22^ A substantial proportion (77/106) of indeterminate AMK results (MTBC-positive) were from smear-negative sputa samples, likely due to low bacillary load or difference detection thresholds.

In summary, our data shows that, while LA-XDR is adept at detecting and ruling in resistance, its potential impact is, like many molecular DSTs, undermined by its ability to detect TB (required to generate a DST result) (**Supplementary Table 6**). For example, in a hypothetical population of 100 smear-negative cases with FQ resistance, 24% (24/100) will be missed.

Our study had limitations, even though we had a large sample size, our cohort had no clinical specimens with LZD resistance, but we included phenotypic-resistant isolates detected by LA-XDR. Using Ultra test results for selection of Group A and B samples introduces variability, and likely could overestimate MTBC sensitivity, but reflects real-world scenarios where LA-XDR would likely be used in many settings. Lastly, we did not evaluate the utility of repeating LA-XDR on sputum, except for false-negative results.

In conclusion, the LA-XDR meets the criteria WHO TPP for a sputum-based next-generation WHO moderate complexity NAAT technology. The LA-XDR can be a valuable tool for MTBC detection and drug susceptibility profiling, especially for LZD and EMB resistance, for which no other commercial molecular DSTs are presently available. Future work should focus on improving the detection of smear-negative TB cases, ensuring that this technology can be effectively utilised in a broader range of clinical scenarios.

## Figures and Tables

**Table 1:** Participant demographic and clinical characteristics. Most participants were men, HIV-negative and culture-negative. Data are % (n/N) or median (IQR).

		Overall (n = 713)	Group A (n = 565)	Group B (n = 87)	Group C (n = 61)
Median (min-max) age (years),		39 (18, 81)	40 (18, 81)	36 (20, 71)	NA
Gender	Male	61 (433/713)	60 (336/565)	63 (55/87)	69 (42/61)
	Female	39 (278/713)	40 (227/565)	37 (32/87)	31 (19/61)
HIV status	Positive	16 (113/713)	14 (80/565)	35 (30/87)	5 (3/61)
	Negative	43 (305/713)	40 (227/565)	23 (20/87)	95 (58/61)
Previous TB		14 (97/713)	13 (72/565)	29 (25/87)	0 (0/61)
Culture-positive		44 (313/713)	29 (165/565)	100 (87/87)	100 (61/61)
Smear-positive		51 (160/313)	39 (65/165)	54 (47/87)	79 (48/61)
Smear-negative		49 (153/313)	61 (100/165)	46 (40/87)	21 (13/61)
FQ resistance		30 (95/313)	0 (0/165)	71 (62/87)	54 (33/61)
EMB resistance		32 (101/313)	0.2 (1/165)	66 (57/87)	71(43/61)
AMK resistance		12 (38/313)	0 (0/165)	25 (22/87)	26 (16/61)
LZD resistance		0 (0/313)	0 (0/165)	0 (0/87)	0 (0/61)

Abbreviation: AMK, amikacin; EMB, ethambutol; FQ, fluoroquinolones; LZD, Linezolid.

Missing data: Gender for Group A (2), HIV (295; Group A, 258; Group B, 37), Age for Group C (61), Previous TB for Group C (61).

**Table 2:** Diagnostic accuracy of LiquidArray MTB-XDR for detecting MTBC from smear-positive and smear-negative sputum samples by DNA extraction method. The sensitivity and specificity are higher in smear-positive than that of smear-negative regardless of DNA extraction methods employed. Data are % (95% CI) and n/N.

	Sputum	
MTBC detection	FL	fleXT
**Overall**		
Sensitivity	87	85
	(82, 91)	(80, 89)
	214/246	199/233
	
		*p=0.528
	
Specificity	98	99
	(97, 99)	(98, 100)
	359/365	337/339
	
		*p=0.278

**Smear-positive**		
Sensitivity	96	94
	(91, 99)	(88, 97)
	105/109	109/115
	
		*p=0.494
	
Specificity	86	100
	(49, 97)	(65, 100)
	6/7	7/7
	
		*p=0.305

**Smear-negative**		
Sensitivity	80	79
	(72, 86)	(71, 85)
	109/137	103/131
	
	^$^ **p<0.001**	*p=0.839
		^$^ **p=0.001**
	
Specificity	99	99
	(97, 99)	(98, 100)
	353/358	330/332
	^$^ **p=0.002**	*p>0.999
		^$^p=0.790

Abbreviation: FL, FluoroLyse; fleXT, GenoXtract fleXT; MTBC, *Mycobacterium tuberculosis* complex.

$ Within-column comparisons between smear statuses

* Within-row comparisons for FluoroLyse vs fleXT.

**Table 3:** Diagnostic accuracy of LiquidArray MTB-XDR for the detection of drug resistance to FQ, AMK, EMB, and LZD compared to composite reference standard by sample type and DNA extraction method. The sensitivity and specificity are higher in isolates than that of sputum regardless of DNA extraction methods employed. The FluoroLyse DNA extraction method is comparable to the GenoXtract fleXT except for the amikacin and ethambutol. Data are % (95% CI) and n/N.

	Sputum		Isolates	
	
Drug susceptibility	FL	fleXT	FL	fleXT
**FQ**				
Sensitivity	93	94	92	92
	(85, 97)	(86, 98)	(85, 96)	(85, 96)
	70/75	66/70	85/92	83/90
	
		*p=0.807	^ɮ^p=0.808	*p>0.999
				^ɮ^p=0.626
	
Specificity	100	99	100	100
	(98, 100)	(96, 100)	(97, 100)	(97, 100)
	166/166	149/150	198/199	199/200
	
		*p=0.197	^ɮ^p>0.999	*p>0.999
				^ɮ^p=0.156

**AMK**				
Sensitivity	64	55	69	72
	(45, 80)	(34, 74)	(54, 81)	(56, 84)
	16/25	11/20	27/39	28/39
	
		*p=540	^ɮ^p=0.678	*p=0.771
				^ɮ^p=0.191
	
Specificity	99	98	100	100
	(96, 100)	(95, 99)	(98, 100)	(99, 100)
	138/139	156/159	255/256	255/255
	
		*p=0.483	^ɮ^p=0.109	*p>0.999
				^ɮ^ **p=0.023**

**EMB**				
Sensitivity	88	85	90	91
	(79, 93)	(75, 91)	(83, 95)	(76, 92)
	78/89	71/84	90/100	90/99
	
		*p=0.563	^ɮ^p=0.660	*p=0.810
				^ɮ^p=0.209
	
Specificity				
	98	97	100	100
	(95, 99)	(94, 99)	(97, 100)	(97, 100)
	166/169	150/154	195/196	194/195
	
		*p=0.564	^ɮ^ **p=0.047**	*p>0.999
				^ɮ^ **p=0.015**

**LZD** ^#^				
Specificity	100	100	100	100
	(97, 100)	(98, 100)	(99, 100)	(99, 100)
	132/132	149/149	289/289	289
	
		*p=^$^	*p=^$^	*p=^$^

Abbreviation: AMK, amikacin; EMB, ethambutol; FQ, fluoroquinolones; FL, FluoroLyse; fleXT, GenoXtract fleXT; LZD, Linezolid; N/A, not applicable.

* Within-row comparisons for FluoroLyse vs fleXT.

ɮ Within-row comparisons for Sputum vs Isolate.

# No LZD resistance samples were evaluated hence sensitivity is incalculable.

$ incalculable.

## References

[1] World Health Organization (2023) Global tuberculosis report 2023. World Health Organization, Geneva

[2] Naidoo P, Theron G, Rangaka MX (2017) The South African Tuberculosis Care Cascade: Estimated Losses and Methodological Challenges. J Infect Dis 216(suppl7):S702–s13 29117342 10.1093/infdis/jix335 PMC5853316

[3] Cox H, Dickson-Hall L, Ndjeka N (2017) Delays and loss to follow-up before treatment of drug-resistant tuberculosis following implementation of Xpert MTB/RIF in South Africa: A retrospective cohort study. PLoS Med ; 14(2). 10.1371/journal.pmed.1002238 PMC5319645 28222095

[4] World Health Organization (2022) WHO Consolidated guidelines on tuberculosis: Module 4: Treatment - Drug-susceptible tuberculosis treatment 35727905

[5] Georghiou SB, de Vos M, Velen K (2023) Designing molecular diagnostics for current tuberculosis drug regimens. Emerg Microbes Infect 12(1):2178243 36752055 10.1080/22221751.2023.2178243 PMC9980415

[6] Theron G, Peter J, Richardson M (2014) The diagnostic accuracy of the GenoType(^®^) MTBDRsl assay for the detection of resistance to second-line anti-tuberculosis drugs. Cochrane Database Syst Rev ; (10): Cd010705 10.1002/14651858.CD010705.pub2 PMC4448219 25353401

[7] Theron G, Peter J, Richardson M, Warren R, Dheda K, Steingart KR (2016) GenoType(^®^) MTBDRsl assay for resistance to second-line anti-tuberculosis drugs. Cochrane Database Syst Rev 9(9):Cd010705 27605387 10.1002/14651858.CD010705.pub3 PMC5034505

[8] David A, de Vos M, Scott L (2023) Feasibility, Ease-of-Use, and Operational Characteristics of World Health Organization-Recommended Moderate-Complexity Automated Nucleic Acid Amplification Tests for the Detection of Tuberculosis and Resistance to Rifampicin and Isoniazid. J Mol Diagn 25(1):46–56 10.1016/j.jmoldx.2022.10.001 PMC9830532 36243289

[9] Kohli M, MacLean E, Pai M, Schumacher SG, Denkinger CM (2021) Diagnostic accuracy of centralised assays for TB detection and detection of resistance to rifampicin and isoniazid: A systematic review and meta-Analysis. Eur Respir J ; 57(2) 10.1183/13993003.00747-2020 32855226

[10] Md Vos, Derendinger B, Dolby T (2018) Diagnostic Accuracy and Utility of FluoroType MTBDR, a New Molecular Assay for Multidrug-Resistant Tuberculosis. J Clin Microbiol 56(9). 10.1128/jcm.00531-18 PMC6113470 29976588

[11] Dippenaar A, Derendinger B, Dolby T (2021) Diagnostic accuracy of the FluoroType MTB and MTBDR VER 2.0 assays for the centralized high-throughput detection of Mycobacterium tuberculosis complex DNA and isoniazid and rifampicin resistance. Clin Microbiol Infect 27(9):1351.e1–.e4 10.1016/j.cmi.2021.04.022 33933566

[12] Denkinger CM, Schumacher SG, Gilpin C (2019) Guidance for the Evaluation of Tuberculosis Diagnostics That Meet the World Health Organization (WHO) Target Product Profiles: An Introduction to WHO Process and Study Design Principles. J Infect Dis10.1093/infdis/jiz097 31593596

[13] Pi R, Liu Q, Jiang Q, Gao Q (2019) Characterization of linezolid-resistance-associated mutations in Mycobacterium tuberculosis through WGS. Journal of Antimicrobial Chemotherapy10.1093/jac/dkz150 31225608

[14] Zhang S, Chen J, Cui P (2016) Mycobacterium tuberculosis Mutations Associated with Reduced Susceptibility to Linezolid. Antimicrob Agents Chemother 60(4):2542–2544 26810645 10.1128/AAC.02941-15 PMC4808145

[15] Derendinger B, Dippenaar A, de Vos M (2023) Bedaquiline resistance in patients with drug-resistant tuberculosis in Cape Town, South Africa: a retrospective longitudinal cohort study. Lancet Microbe 4(12):e972–e82 37931638 10.1016/S2666-5247(23)00172-6 PMC10842724

[16] FIND, Biobank services - (2023) FIND. https://www.finddx.org/what-we-do/cross-cutting-workstreams/biobank-services/ (accessed 2023-02-02

[17] Bruker-HainLifescience (2022) Fluoro Type ^®^ XDR VER 1.0 (β-Version) PCR Kit, vol 0. Bruker-Hain Lifescience GmbH, pp 1–26

[18] World Health Organization (2022) Optimized broth microdilution plate methodology for drug susceptibility testing of Mycobacterium tuberculosis complex

[19] Streicher EM, Bergval I, Dheda K (2012) Mycobacterium tuberculosis population structure determines the outcome of genetics-based second-line drug resistance testing. Antimicrob Agents Chemother 56(5):2420–2427 22330913 10.1128/AAC.05905-11 PMC3346650

[20] Victor TC, Jordaan AM, van Rie A (1999) Detection of mutations in drug resistance genes of Mycobacterium tuberculosis by a dot-blot hybridization strategy. Tuber Lung Dis 79(6):343–348 10694978 10.1054/tuld.1999.0222

[21] Zimenkov DV, Nosova EY, Kulagina EV (2017) Examination of bedaquiline- and linezolid-resistant Mycobacterium tuberculosis isolates from the Moscow region. J Antimicrob Chemother 72(7):1901–1906 28387862 10.1093/jac/dkx094

[22] World Health Organization (2023) Catalogue of mutations in Mycobacterium tuberculosis complex and their association with drug resistance, 2nd ed

[23] World Health Organization (2021) In: Organization WH (ed) Update on the use of nucleic acid amplification tests to detect TB and drug-resistant TB: rapid communication. World Health Organization

[24] Pillay S, Davies GR, Chaplin M (2021) Xpert MTB/XDR for detection of pulmonary tuberculosis and resistance to isoniazid, fluoroquinolones, ethionamide, and amikacin. Cochrane Database of Systematic Reviews ; 2021(6) 10.1002/14651858.CD014841.pub2 PMC9115865 35583175

[25] Penn-Nicholson A, Georghiou SB, Ciobanu N (2022) Detection of isoniazid, fluoroquinolone, ethionamide, amikacin, kanamycin, and capreomycin resistance by the Xpert MTB/XDR assay: a cross-sectional multicentre diagnostic accuracy study. Lancet Infect Dis ; 22(2) 10.1016/S1473-3099(21)00452-7 34627496

[26] World Health Organization (2014) High priority target product profiles for new tuberculosis diagnostics: report of a consensus meeting, 28–29 April 2014, Geneva, Switzerland

[27] Maningi NE, Malinga LA, Antiabong JF, Lekalakala RM, Mbelle NM (2017) Comparison of line probe assay to BACTEC MGIT 960 system for susceptibility testing of first and second-line anti-tuberculosis drugs in a referral laboratory in South Africa. BMC Infect Dis 17(1):795 29282012 10.1186/s12879-017-2898-3 PMC5745758

[28] Beckert P, Hillemann D, Kohl TA (2012) rplC T460C identified as a dominant mutation in linezolid-resistant Mycobacterium tuberculosis strains. Antimicrob Agents Chemother 56(5):2743–2745 22371899 10.1128/AAC.06227-11 PMC3346602

